# Single-Use Capture Purification of Adeno-Associated Viral Gene Transfer Vectors by Membrane-Based Steric Exclusion Chromatography

**DOI:** 10.1089/hum.2019.284

**Published:** 2021-09-23

**Authors:** Pavel Marichal-Gallardo, Kathleen Börner, Michael M. Pieler, Vera Sonntag-Buck, Martin Obr, David Bejarano, Michael W. Wolff, Hans-Georg Kräusslich, Udo Reichl, Dirk Grimm

**Affiliations:** ^1^ Bioprocess Engineering, Max Planck Institute for Dynamics of Complex Technical Systems, Magdeburg, Germany.; ^2^ Center for Infectious Diseases, Virology, Heidelberg University Hospital, Heidelberg, Germany.; ^3^ BioQuant, Heidelberg University, Heidelberg, Germany.; ^4^ German Center for Infection Research (DZIF), Partner Site Heidelberg, Heidelberg, Germany.; ^5^ Institute of Bioprocess Engineering and Pharmaceutical Technology, Technische Hochschule Mittelhessen, Gießen, Germany.; ^6^ Cluster of Excellence CellNetworks, Heidelberg, Germany.; ^7^ Bioprocess Engineering, Otto von Guericke University Magdeburg, Magdeburg, Germany.; ^8^ German Center for Cardiovascular Research (DZHK), Partner Site Heidelberg, Heidelberg, Germany.

**Keywords:** membrane chromatography, virus purification, downstream processing, polyethylene glycol, regenerated cellulose, AAV

## Abstract

We present membrane-based steric exclusion chromatography (SXC) as a universal capture step for purification of adeno-associated virus (AAV) gene transfer vectors independent of their serotype and surface characteristics. SXC is performed by mixing an unpurified cell culture supernatant containing AAV particles with polyethylene glycol (PEG) and feeding the mixture onto a chromatography filter unit. The purified AAV particles are recovered by flushing the unit with a solution lacking PEG. SXC is an inexpensive single-use method that permits to concentrate, purify, and re-buffer AAV particles with yields >95% and >80% impurity clearance. SXC could theoretically be employed at industrial scales with units of nearly 20 m^2^.

## Introduction

Adeno-associated virus (AAV)-based gene therapy offers the prospect of treating a wide range of diseases, such as cancer,^1,2^ hemophilia,^3^ Duchenne muscular dystrophy,^4^ and vision loss.^5,6^ The promise and potential of this technology is perhaps best exemplified by the market authorization of three commercial gene therapy products derived from wild-type AAV serotypes, that is, Glybera (AAV1), Luxturna^®^ (AAV2) and, most recently, Zolgensma^®^ (AAV9). While these success stories are highly encouraging and motivate a strongly increasing number of academic and industrial entities to enter the field of AAV vector engineering and application, they concurrently raise a demand for advanced technologies for AAV manufacturing and purification at various process scales.

Typically, recombinant AAV vectors are produced in mammalian or insect cells and then recovered from cell lysates, although some AAV variants may also accumulate in, and be recovered from, cell culture supernatants.^7^ For research and development purposes, AAV particles are often purified using density gradient ultracentrifugation with isopycnic cesium chloride (CsCl) gradients or iodixanol step gradients. These methods can achieve high purity and largely eliminate undesired empty AAV capsids (∼20% remaining with iodixanol and <1% with CsCl), but density gradient ultracentrifugation has several disadvantages. For instance, it is rather time-consuming (up to 3 h for iodixanol, and even up to 36 h for CsCl) and involves substantial manual work that is difficult to automate. Moreover, the required centrifuges are expensive, product yields are low (often <20%), and processing of large volumes or of multiple samples is challenging.^6,8–11^ Whereas iodixanol is a contrast agent used in humans, CsCl is a cytotoxic compound that has to be removed from the final vector preparation, for example, by dialysis, resulting in further vector losses. Finally, both methods can require several rounds to achieve the desired degree of purity, which multiplies time, labor, and costs.^12^ Consequently, there is an urgent and increasing need for new technologies that can complement or entirely replace the use of density gradient ultracentrifugation for AAV manufacturing at the laboratory and industrial scale.^9,10,13,14^

Indeed, other methods have been developed and applied for AAV purification that comply with current good manufacturing practice and that are typically based on chromatography techniques.^15^ One notable variant is ion exchange chromatography (IEX) that can separate full and empty AAV capsids and has already been used for purification of several AAV serotypes comprising AAV2, AAV4, AAV5, and AAV8.^16–19^ However, the IEX process is not universally applicable but rather needs to be adapted and optimized for each AAV capsid variant.

Another option is heparin affinity chromatography that can be used to purify certain AAV serotypes, such as AAV2 or AAV6, based on their natural affinity to heparan sulfate proteoglycans. Alas, only a few AAV serotypes and variants share this specific affinity and are thus amenable to this purification method.^8^ The same restriction hampers the wider use of pseudo-affinity chromatography with sulfated resins, such as Cellufine^®^ Sulfate or sulfated membrane adsorbers.^20^

A recent and very intriguing option is the use of affinity matrices based on 14 kDa camelid antibodies that can reliably and robustly capture AAV particles. For example, product yields of 50–92% were reported for the AVB Sepharose™ resin when used to purify AAV1, AAV2, AAV5, or AAV6.^21^ While AAV8 and AAV9 bind poorly to this particular resin, they are compatible with newer resins that fill in this gap, such as POROS™ CaptureSelect™ AAV8 and AAV9. Most recently, a POROS™ CaptureSelect™ AAVX resin was introduced that can be used to purify serotypes AAV1 through AAV9 as well as several chimeric and recombinant capsid variants.^14^

Despite the many advantages of affinity chromatography, this technology also presents several drawbacks. These include the need for careful optimization of wash and elution conditions for each individual product, as well as the use of acidic buffers for elution, which might induce product losses or adverse particle aggregation. Furthermore, affinity chromatography cannot separate empty and full capsids, which necessitates at least one subsequent purification step. In addition, ligand leaching can pose problems. Moreover, the high cost of affinity resins hampers their single-use operation, requiring cleaning and sanitizations steps that in turn add to process development and AAV manufacturing costs. Finally, bead-based chromatography processes require packaging and validation of columns, and flow rates are low compared with methods that utilize stationary phases such as membranes and monoliths.

Here, we focus on a purification method that is called “steric exclusion chromatography” (SXC) and that exploits molecular crowding effects caused by the addition of a “crowding agent” (*e.g.*, polyethylene glycol [PEG]) to a solution.^22^ In SXC, a crude sample containing the target species is first mixed with PEG, and the product is then captured without a direct chemical interaction on a nonreactive hydrophilic surface. The product is finally recovered by reducing the PEG concentration in the mobile phase. Selectivity in SXC is highly correlated with the hydrodynamic size of the target product, with larger molecules more prone to interact with the stationary phase than smaller ones, which makes this method particularly well suited for the purification of virus particles. A major benefit of SXC is that the target product is loaded and recovered at physiological pH and salt concentration. This circumvents the use of process conditions that might damage or reduce the biological activity of the product, such as acidic pH or low/high salt concentrations that might induce virus aggregation. Moreover, PEG is an inert substance that is known to increase the stability of protein structures.^23,24^ Recently, we have shown that membrane-based SXC using inexpensive cellulose membranes is an efficient and single-use purification method that can be applied for purification of a wide variety of virus particles, such as influenza virus^22^ and yellow fever virus.^25^ Remarkably, different strains could be purified using the same chromatography conditions, with product yields exceeding 95%.

In the present protocol, we describe, for the first time, the use of membrane-based SXC as an efficient method for the purification of AAV vectors with self-made filter units packed with disposable cellulose membranes of 1.0 μm pore size. We characterize the established workflow by documenting the transduction or knockdown efficiency of purified vectors, and present data regarding the recovery and purity of several AAV serotypes and synthetic capsid variants purified using identical conditions.

Importantly, SXC can be used to inexpensively and quickly concentrate large volumes of cell culture supernatant that would be challenging with other methods and that are otherwise discarded. Additionally, this method can also be performed manually without the need of a chromatography system. We conclude that SXC has the potential to become a platform approach for the initial capture of AAV particles independent of their surface characteristics and that can be used to complement and expand existing purification processes.

## Materials

### Reagents and supplies

All the materials are listed in Table 1. For all solutions, deionized water was used, referred to as “water” hereafter. All concentrations are expressed as percentages (%) or mass/volume (m/v) unless stated otherwise. All chemicals used had a purity of *≥*99% unless noted otherwise. Cells from human donors (*i.e.,* monocyte-derived macrophages) were obtained according to the regulations of the local ethics committee of the Heidelberg University Hospital.

**Table 1.** Materials (reagents, supplies, and biologics)

Material	Supplier	Cat. no.	Specific handling	Storage
Reagents				
DMEM + GlutaMAX	Invitrogen/Gibco	61965		4°C
FBS	Biochrom AG	S 0115		−20°C
Penicillin–streptomycin	Invitrogen/Gibco	Various		−20°C
Liquid nitrogen	Various			
TurboFect transfection reagent	Thermo Scientific	R0531		4°C
PEG-6000	Sigma–Aldrich	81260		RT
Sodium hydroxide	Sigma–Aldrich	S7653	Corrosive	RT
Hydrochloric acid 37%	Sigma–Aldrich	320331	Corrosive	RT
Sodium chloride	Sigma–Aldrich	S8045		RT
PEI	Polyscience	23966		RT
PFA	Electron Microscopy Science	15710		RT
Hoechst 33258	ThermoFisher Scientific	H1398		4°C
PTA	Serva	32757.01		RT
Pioloform BM18	Plano GmbH	R1275B		
Quant-IT™ DNA assay kit	ThermoFisher Scientific	Q33210		4°C
Quant-IT protein assay kit	ThermoFisher Scientific	Q33120		4°C
ddPCR Supermix for probes	Bio-Rad	186310		−20°C
Supplies				
Bottle top filter 0.2 μm	VWR	514-0340		RT
Syringe filters 0.8, 0.65, 0.45, and 0.2 μm	Sartorius Stedim Biotech	Various		RT
Regenerated cellulose membranes 1.0 μm	Cytiva	10410014		RT
Stainless steel filter holder 25 mm	Merck Millipore	XX3002500		RT
Superdex 200 Increase 10/300 GL column	Cytiva	17517501		RT
Rubber O-ring	Cytiva	18-1029-60		RT
Hole puncher (25 mm) for regenerated cellulose membranes	Various	DIN 7200 A		RT
1.5–2.0 mL centrifuge tubes	Various			RT
15 and 50 mL centrifuge tubes	Various			RT
Disposable syringes, 10–20 mL	Various			RT
Cell lifter, high-density polyethylene	Corning	3008		RT
15 cm dishes	Various			RT
6-well plates	Various			RT
Flasks 75 cm^2^ (T75)	Various			RT
96-well flat bottom assay plate, black polystyrene	Corning	3915		RT
250 mL centrifuge tubes	Sigma-Aldrich	CLS430776		RT
Whatman grade 1 filter paper	Cytiva	1001-110		RT
Twin.tec 96-well PCR plate	Eppendorf AG	Various		
Dialysis membranes, 100 or 300 kDa cutoff	Spectrum	131414 or 131450		4°C
300 mesh copper grids	Plano GmbH	G2430C		RT
Biologics				
HEK293T cells	ATCC	CRL-3216		−80°C
AAV helper plasmid (encoding the cap gene of AAV1, AAV2, or AAV6)	Ref.^21^			−20°C
Benzonase^®^ or Denarase^®^ endonuclease	Merck Millipore or c-LEcta			−20°C

AAV, adeno-associated virus; ddPCR, Droplet Digital™ polymerase chain reaction; DMEM, Dulbecco's modified Eagle medium; FBS, fetal bovine serum; PEG, polyethylene glycol; PEI, polyethylenimine; PFA, paraformaldehyde; PTA, phosphotungstic acid; RT, room temperature.

### Equipment

(1) ÄKTA fast protein liquid chromatography (FPLC) system with fraction collector (Cytiva)
(2) Inverted microscope IX81 (Olympus Biosystems)
(3) Electron microscope EM10 operated at 80 kV (Zeiss)
(4) High vacuum coater EM ACE600 (Leica)
(5) Plate reader Safire (Tecan)
(6) QX200 Droplet Reader (Bio-Rad Laboratories)
(7) PX1 PCR Plate Sealer (Bio-Rad Laboratories)
(8) C1000 Touch Thermal Cycler (Bio-Rad Laboratories)
(9) Water bath
(10) Vortex mixer
(11) Incubator
(12) Refrigerator
(13) Freezer
(14) Glow discharging unit

### Reagent setup

(1) Phosphate-buffered saline (PBS) stock (20 × ): dissolve 2.88 g of Na_2_HPO_4_, 160 g of NaCl, 4.8 g of KH_2_PO_4_, and 4.0 g of KCl in water. Sterilize by filtration.
(2) Cell fixing solution: 4% (v/v) paraformaldehyde (PFA) in 1 × PBS.
(3) Cell staining solution: 330 ng/mL of Hoechst 33258 in 1 × PBS.
(4) Cell culture medium: Dulbecco's modified Eagle medium (DMEM) supplemented with 10% (v/v) fetal calf serum, 100 U/mL penicillin, and 100 μg/mL streptomycin.
(5) Two percent phosphotungstic acid (PTA): prepare a 2% solution of PTA in water. Adjust pH to 7.5 with NaOH.
(6) 1.2% Pioloform in chloroform: dissolve 0.6 g of Pioloform in 50 mL of chloroform.
(7) Lysis buffer (50 mM Tris-HCl, 150 mM NaCl, 2 mM MgCl_2_, pH 8.5): dissolve 7.88 g of Tris-HCl, 8.77 g of NaCl, and 190.42 mg of MgCl_2_ in 900 mL of water. Adjust pH to 8.5 and complete to 1 L with water. Sterilize by 0.2 μm filtration.
(8) Thirty-two percent PEG-6000 (PEG stock): prepare 1 L of a stock of 32% PEG-6000 (molecular mass 6,000 Da) by weighing 320 g of PEG-6000 and filling up to 1 L. Sterile filter with a 0.2 μm bottle top filter in 250 mL bottles and store at room temperature (RT) for up to 2 months.

(a) Alternatively, a 20% PEG-6000, 1 × PBS solution can be used for a 1:1 in-line dilution of the sample when using an automated chromatography system with a binary pump. The in-line dilution is recommended to reduce the risk of aggregation and to increase binding capacity during SXC (further discussion in the “Results” section). The in-line dilution approach also allows for flexibility with the amount of sample to be purified since the loading can be stopped and the remaining unpurified sample stored.

(9) Binding buffer (10% PEG-6000, 1 × PBS): prepare 1 L of binding buffer with a 10% PEG-6000 concentration by mixing 50 mL of PBS stock with 312.5 mL of PEG stock and filling up to 1 L. Sterile filter with a 0.2 μm bottle top filter in 500 mL bottles and store at 4°C for up to 2 months.
(10) Elution buffer (1 × PBS): prepare 500 mL of elution buffer by mixing 25 mL of PBS stock with 475 mL of water. Sterile filter with a 0.2 μm bottle top filter in 250 mL bottles and store at 4°C for up to 2 months.

(a) Note: Both binding and elution buffers can be based on commonly used buffer systems that are suitable for AAV particles, for example, PBS or Tris-HCl buffer.

(11) Cleaning-in-place (CIP) buffer (1.0 N NaOH, 2.0 M NaCl): prepare 1 L of CIP buffer by dissolving 40 g of NaOH in 800 mL of water. Afterward, add 117 g of NaCl and fill up to 1 L. Filter through a 0.45 μm (or less) filter and store at RT for up to 2 months.

(a) Note: NaOH is highly corrosive. Always use eye protection and take special care when using it with pressurized systems (*e.g.*, the chromatography system).

## Experimental Procedure

**Figure 1.**
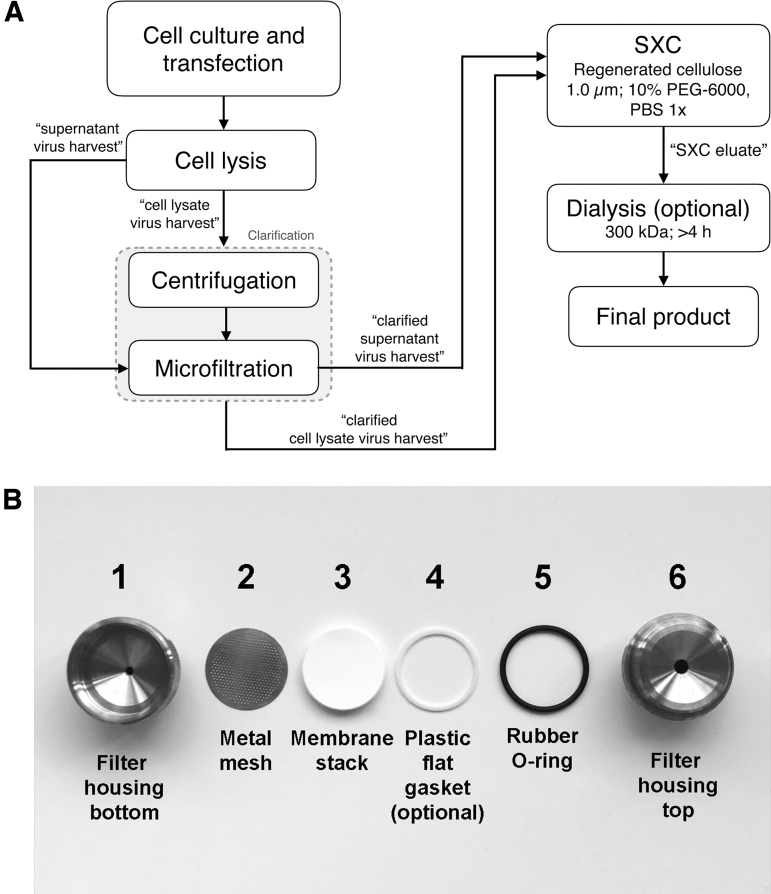
**(A)** Experimental procedure (see Table 2 for timing of all steps including analytics). **(B)** Assembly of the SXC filter unit. SXC, steric exclusion chromatography.

An overview of the workflow is shown in Fig. 1A.

### AAV vector stock production—small scale

(1) Produce small-scale AAV vector stocks (“crude lysates”) in 6-well plates by seeding 3.5 × 10^5^ HEK293T cells per well in 4 mL DMEM and by incubating them at 37°C and 5% CO_2_ for 24 h.
(2) Prepare the transfection mixture in 390 μL DMEM without any supplements by adding equal amounts (1.3 μg per plasmid, totaling 4 μg of DNA) of the AAV helper plasmid^26^ encoding AAV *rep*, *cap*, and *aap* genes, the adenoviral helper plasmid, and the AAV vector construct. In the examples shown below, the latter encoded a yellow fluorescent protein (YFP) reporter cDNA driven by the cytomegalovirus (CMV) promoter.
(3) Add TurboFect transfection reagent (8 μL per well), vortex the transfection solution, and incubate for 15 min at RT.
(4) Carefully distribute 400 μL of the mixture per well and incubate the cells at 37°C for 72 h.
(5) Scrape off the cells and transfer them to 15 mL tubes, before concentrating them by centrifugation at 1,500 relative centrifugal force (rcf) for 15 min. Remove the supernatant, resuspend the cells in 300 μL PBS, and transfer into 2 mL tubes. The removed cell supernatant contains AAV particles and can be directly processed as described in the “Sample Preparation for SXC” section for purification with SXC.
(6) Subject the samples to five cycles of freezing and thawing (5 min each) using a 37°C water bath and liquid nitrogen. This sample is called “virus harvest” hereafter.
(7) Centrifuge the lysed solution at a minimum of 10,000 rcf for 10 min to remove cell debris.
(8) Store the supernatant containing the virus particles (centrifuged virus harvest) at −80°C in 50 μL aliquots.

### AAV vector stock production—medium scale

(1) Produce medium-scale AAV vector stocks in 15 cm dishes by seeding 4.5 × 10^6^ HEK293T cells per well in 22 mL DMEM and by incubating them at 37°C and 5% CO_2_ for 48 h.
(2) Prepare the transfection mixture in 4 mL DMEM without any supplements by adding equal amounts of the three plasmids (14.6 μg per plasmid, in total 43.8 μg of DNA) as for the small scale above. The amounts are given for one 15 cm dish and can be expanded.
(3) Add 140 μL polyethylenimine (PEI) (1 mg/mL) transfection reagent for each plate to the master mix, vortex the transfection solution, and incubate for 30 min at RT.
(4) Carefully distribute 4 mL of the mixture per dish and incubate the cells at 37°C for 72 h.
(5) Scrape the cells from the dishes, pooling at least five dishes per AAV construct, and centrifuge for 20 min at 1,500 rcf. Remove the supernatant and resuspend the cell pellet in 6 mL of virus lysis buffer. The removed cell supernatant contains AAV particles and can be directly processed as described in the “Sample Preparation for SXC” section for purification with SXC.
(6) Subject the samples to five cycles of freezing and thawing (5 min each) using a 37°C water bath and liquid nitrogen.
(7) Sonicate the sample for 1 min 20 s.
(8) Add 75 U/mL Benzonase to the virus-containing solution and keep the sample for 1 h at 37°C, inverting it every 10 min.
(9) Centrifuge the lysed solution twice for 15 min at 4,000 rcf, 4°C to remove cell debris.
(10) Store the solution at −20°C in 50 mL Falcon tubes.

### Sample preparation for SXC

(1) Clarify the centrifuged virus harvests (cell lysate and supernatant) by microfiltration (cellulose acetate filters are appropriate, Table 1). A final pore size of 0.2 μm is recommended. In case a 0.2 μm filter is blocked with the centrifuged material from step (7) described in the “AAV Vector Stock Production—Small Scale” section, the blockage can be reduced and the risk of product losses due to clogged filters is minimized by using a cascade of filters (*e.g.*, 0.8, 0.45, and 0.2 μm is often appropriate). The filtered sample is named “clarified cell lysate virus harvest” and “clarified supernatant virus harvest” hereafter (Fig. 1A).
(2) Add the required amount of PEG stock to the clarified virus harvest to achieve the final PEG concentration of 10% needed to capture the AAV particles during SXC. Use the following formula to calculate the needed volume of PEG stock:

$$V_{PEG_{stock}}=\frac{\%PEG_{feed}\cdot V_{feed}}{32\%}.$$

- (a) Example: for preparing 10 mL of PEG-conditioned sample at 10% PEG-6000:
- *V*_feed_ = 10 mL
- %PEG_feed_ = 10%
- $V_{PEG_{stock}}$ = 10* ×* 10/32 = 3.125 mL
(i) In this example, the volume of clarified virus harvest to be purified has to be equal or lower than 10 mL−3.125 mL = 6.875 mL. The clarified virus harvest can be diluted with 1 × PBS to reach this volume.
- (b) Example: if using a chromatography system with a binary pump, a 1:1 in-line dilution of the clarified virus harvest with 20% PEG-6000, 1 × PBS is highly recommended over conditioning the sample with PEG off-line (further discussion in the “Results” section).

### Assembly of SXC filter unit

This filter unit can be used for either purification using a chromatography system (the “SXC Using a Liquid Chromatography System” section) or manual purification with a syringe (the “SXC Performed Manually with a Syringe” section). The filter unit is alternatively called a column. Assemble the column components in the order stated in Fig. 1B:

(1) Stack up to 20 membranes (100 cm^2^) and place them on top of the stainless steel mesh inside the filter housing.
(2) Add a plastic flat gasket on top of the membranes (optional). The plastic gasket is meant for the very rare situation in which a part of the membrane pops out from the inner diameter of the rubber O-ring while screwing the device. This can cause the fluid to flow through the filter unit without touching the membranes, leading to product loss during purification. This optional gasket helps keep the membranes in place. Additionally, a second metal mesh can be placed on top of the membranes instead of the flat gasket.
(3) Add the O-ring. A rubber O-ring is recommended because it is flexible and it seals the device better compared with a rigid gasket.
(4) Screw the top part of the housing without overtightening. With the materials stated here and a stack of 20 membranes, a tightened assembled column with a height of *≤*17.60 mm is unlikely to leak. However, the column can be tested for leaks by flushing it with 5–10 mL of binding buffer using a syringe at a speed of 5–10 mL/min or directly in the chromatography system.

(a) Note: The experimenter can also use filter housings with a smaller diameter, for example, 14 mm at the expense of lower binding capacity and slightly more back pressure during purification, which is especially noticeable when purifying samples manually with a syringe.
(b) Note: Teflon tape can be used for sealing the thread of the housing to reduce the risk of leaks and to protect the screw, which can be damaged over time (*e.g.*, due to tightening without proper aligning).
(c) Although the bed volume of the compressed stack of twenty 2.5 cm membranes is around 0.4 mL, for practical purposes, the column volume (CV) can be defined as 1 mL.

### SXC using a liquid chromatography system

SXC with a chromatography system is always recommended over a manual purification. A FPLC system provides controlled flow rates, pressures, the possibility to perform gradients, online monitoring of process variables such as ultraviolet (UV) absorbance and conductivity, and precise sample fractionation. This general workflow can be carried out with any liquid chromatography system once it has been adapted to each specific equipment. The recommended flow rates shown here are for an ÄKTA Pure 25 FPLC system equipped with two pumps. The total protein is monitored online by UV absorbance at 280 nm. Optionally, a light-scattering detector coupled to the chromatography system can be used to trace the virus particles.

#### Preparation of the liquid chromatography system

(a) Connect the purification column to the system.
(b) Water wash: flush the entire system with water.
(c) Priming of buffer inlets: prime inlet B with elution buffer and inlet A with binding buffer (flow rate: 10 mL/min).
(d) Column equilibration: wash with at least 10 CV of water, followed by at least 10 CV of binding buffer (flow rate: 10 mL/min). Check for any leaks and allow the system to achieve baseline UV, pressure, and conductivity.

#### SXC purification of AAV particles

A predefined method can be harnessed to run purifications using a chromatography system. Most devices provide ready-to-use templates. For the SXC purification described here, a generic affinity purification template with slight modifications is acceptable. The filter unit can withstand a pressure of 2.0 MPa. The duration of the steps in a method may be programmed based on CV (1 mL for the purification column described here), volume, or time. Here, we express the duration in volume (mL). The method run should have the following steps:

(a) Column equilibration: flush the column with at least 10 mL of binding buffer.
(b) Sample injection (in-line dilution with 20% PEG-6000, 1 × PBS is recommended): inject the sample to the column at a flow rate up to 10 mL/min. Collect the flow-through as a whole or in desired volume fractions.
(c) Column wash: flush the column with at least 15 mL of binding buffer or until UV signal is stable. Collect the wash as a whole or in desired volume fractions.
(d) Elution: recover the product by flushing the column with elution buffer at 5 mL/min. Collect fractions of 0.5–1 mL. To maximize product recovery, collect at least 10 mL.
(e) CIP (optional): the low cost of the membranes allows this operation to be single use. It is thus recommended to dispose the membranes after each purification to avoid performance loss and the risk of impurity carryover between runs. From our experience, however, the membranes can be reused after proper cleaning without noticeable effects on product quality. Nevertheless, the degree to which the membranes can be reused has to be assessed by the experimenter for each particular sample. If a purification column is reused, it should be restricted to a single vector type and at best only for replicated purifications. Flush the column with 15 mL of CIP buffer and let it rest for 15 min. Flush with 20 mL of water. At this point, the column can be re-equilibrated with binding buffer for another purification run or flushed with 10 mL of 20% (v/v) ethanol for storage.

### SXC performed manually with a syringe

SXC can also be performed manually without a chromatography system. Besides situations where there is no chromatography system available, performing SXC manually with disposable syringes is recommended for quick purifications when there is no need to monitor variables such as UV absorbance in real time or when precise fractionation is not required. Beware that manual purifications are performed at the expense of losing control over many process variables.

(1) Column equilibration: flush the column with at least 10 mL of binding buffer.
(2) Sample injection: apply the sample to the column at a speed of approximately one drop per second. Resistance to the flow is normal and is due to the column, the sample, and the viscosity from the PEG. Collect the flow-through as a whole or in desired volume fractions.
(3) Column wash: flush the column with at least 15 mL of binding buffer. Collect the wash as a whole or in desired volume fractions.
(4) Elution: flush the column with 10–15 mL of elution buffer at a lower speed than used for sample injection and washing. Collect eluate in 0.5–1 mL fractions.

(a) Note: After a few drops with the elution buffer, pressure will suddenly decrease and it will be easier to flush the column. This decrease in pressure happens because of the lower viscosity of the elution buffer and the detachment of the virus particles from the column.

### Dialysis of purified AAV particles (optional)

Dialysis of the purified AAV can be performed after SXC to exchange the sample buffer, eliminate traces of PEG, and for desalting if the sample will be lyophilized and resuspended in a lower volume. Molecular mass cutoffs above 50 kDa are recommended to fully eliminate traces of PEG that might remain in the samples with a pore size of 14 kDa or less. Using 300 kDa is standard practice for AAV particles, even for concentration/diafiltration steps at industrial scale with cross-flow filtration.

(1) Rinse the dialysis membranes in a beaker with water.
(2) Prepare the dialysis solution with the buffer of choice. A sample-to-buffer ratio of 1:1,000 is recommended and 1:200 is sufficient.
(3) Place the sample inside the dialysis tubes and seal them tight.
(4) Start dialysis under stirring, at best at 4°C. With pore sizes of 100 kDa and above, 4 h are typically sufficient for a full buffer exchange with a 1:1,000 sample-to-buffer ratio, but the dialysis can be performed overnight.

(a) As a general rule, AAV particles can be formulated in 1 × PBS, 350 mM NaCl, and 5% (v/v) sorbitol (or glycerol).^6,27^
(b) Particle aggregation of AAV can be minimized by using divalent ion salts, for example, 200 mM magnesium sulfate. Non-ionic surfactants such as Pluronic F68 at a concentration of 0.001% (v/v) can also be used.^14^

### Reporter activity assay to monitor *in vitro* transgene expression in cells transduced with SXC-purified AAV

Cells are fed with the SXC-purified AAV particles, and the transduction rates and mean fluorescence intensities of the YFP/GFP reporter-encoding AAV vector are determined by microscopy.

(1) Transduce susceptible cells (*e.g.*, HEK293T, SF539, U87, or monocyte-derived macrophages) with SXC-purified AAV particles at different dilutions in a total volume of 80 μL per well in a 96-well plate. Incubate the cells for 36–48 h at 37°C and 5% CO_2_ in a humidified atmosphere.
(2) Fix the cells with 80 μL per well of fixing solution at RT for 30 min, and afterward, treat the cells with Hoechst staining solution to label nuclei. For immunofluorescence analysis, use respective primary and secondary antibody combinations. In the following, the procedure used for the examples shown in Fig. 5 is described. Incubate cells for 10 min with PBS containing 0.2% Triton X-100, followed by 30 min incubation with 5% fetal bovine serum (FBS) in PBS. Dilute the primary antibody according to the datasheet in 5% FBS in PBS and incubate for 2 h at RT or overnight at 4°C. Wash the cells three times with PBS for 5 min each. Add Alexa Fluor-labeled secondary antibody (1:1,000 diluted in 5% FBS) and counterstain with Hoechst for 2 h at RT in the dark. Wash the cells three times with PBS.
(3) Use a fully automated microscope for image acquisition, such as the Olympus Biosystems IX81 microscope.
(4) Acquire images in the Hoechst and in the reporter channel with a 10 × objective in nine different positions.
(5) Perform an automated image analysis consisting of three successive steps: (i) cell nuclei segmentation in the Hoechst channel, (ii) cell segmentation in the reporter channel, and subsequent (iii) grey value quantification of the identified objects. Automated image analysis is performed to determine the reporter mean intensities and to classify transduced and nontransduced cells, as well as to determine the mean intensity of target proteins after AAV-mediated knockdown. Further details of the entire workflow are published elsewhere.^26^

### Determination of AAV genomes via Droplet Digital™ PCR

Sample preparation via alkaline lysis of AAV particles:

(1) Incubate AAV lysates with 50 U/mL Benzonase for 1 h at 37°C.
(2) Spin down cell debris by centrifugation at 3,750 rcf for 20 min.
(3) For alkaline lysis of AAV capsids, dilute the samples 1:2 in TE (DNA/DNase-free) to a final volume of 20 μL.
(4) Add 20 μL of 2 M NaOH to the previous sample and mix well.
(5) Incubate sample for 30 min at 56°C.
(6) Stop the alkaline lysis by adding 38 μL of 1 M HCl.
(7) Dilute samples further to 1 mL with water, followed by an additional dilution step (1:100) with water.

- (a) Primers and probe are directed against the YFP/GFP sequence.
(i) FAM-ACGACGGCAACTACA-BHQ1, primer forward 5′-GAGCGCACCATCTTCTTCAAG-3′ and primer reverse 5′-TGTCGCCCTCGAACTTCAC-3′.
- (b) For droplet generation, prepare a reaction mix containing:
(i) 0.99 pmol of each primer, 0.275 pmol of probe, and 11 μL of 2 × ddPCR (Droplet Digital PCR) supermix for probes (Bio-Rad Laboratories).
(i i) Add 2.2 μL of water and 5.5 μL of sample.

ddPCR: droplets are generated with a QX200 Droplet Generator using droplet generation oil for probes (all Bio-Rad Laboratories).

(8) Transfer droplet-containing samples to a Twin.tec 96-well PCR plate (Bio-Rad Laboratories) and seal it with Foil Seals for PCR and QX200 ddPCR application (Bio-Rad Laboratories) using the PX1 PCR Plate Sealer (Bio-Rad Laboratories).
(9) Carry out the PCR in a C1000 Touch Thermal Cycler using the following protocol with the ramp rate set to 2°C per cycle:

**Table:** 

Temperature (°C)	Time	
94	10 min	
94	30 s	40 ×
60	60 s	
98	10 min	
12	Hold

(10) Subsequently, droplets are subjected to a QX200 Droplet Reader (Bio-Rad Laboratories).
(11) Analyze the raw data with the corresponding software QuantaSoft 1.7 operating in combined well analysis mode.

### Evaluation of impurity depletion of SXC-purified AAV particles

Follow the manufacturer's instructions for the total protein assay and the double-stranded DNA (dsDNA) assay kits listed in Table 1. Construct a calibration curve that fits the measured data. Beware that proper regression analysis has to be made to avoid the calculation of misleading results. Follow the recommendations of Ellison *et al.* for regression analysis and statistical validation.^28^ As an alternative to the dsDNA and total protein quantification methods described here, we refer the reader to Marichal-Gallardo *et al*.^22^

Additionally, perform sodium dodecyl sulfate–polyacrylamide gel electrophoresis (SDS-PAGE) with Coomassie or silver staining. The viral proteins VP1/2/3 can be detected by Western blot (WB) with B1 antibody.

### Evaluation of physical integrity of SXC-purified AAV by negative staining and transmission electron microscopy

For the transmission electron microscopy (TEM) analysis, use 300 mesh copper grids coated with 1.2% Pioloform and 3 nm carbon film, or similar.

(1) Pioloform coating of the electron microscopy (EM) grids: place a clean microscopic glass slide into a cylindrical separating funnel of a suitable size, so that the slide is standing upright on the bottom of the funnel. Fill the funnel with 1.2% Pioloform solution to *≈*2/3 of the glass slide height. Release the Pioloform solution from the funnel. In a clean vessel with water, slowly float the Pioloform film from the glass slide onto the water surface. Lay the grids onto the film and avoid grids overlapping each other. Pick up the film with grids by a strip of parafilm. Let the grids air-dry for at least 1–2 days.
(2) Carbon coating of the EM grids: coat the grids on the parafilm strip with a 3 nm thick layer of carbon in the Leica EM ACE600 (or equivalent carbon coater) according to the manufacturer's instructions.

(a) Note: If the grids are precoated by the manufacturer, skip steps 1 and 2 and proceed directly to step (3).

(3) Glow-discharging of the coated EM grids: before sample application, glow-discharge the grids to be used for 30 s in Pelco easiGlow (or equivalent glow-discharging unit).
(4) Sample application and wash: apply 3–5 μL of the sample to the grid for 5 min. Wash the grid twice by briefly inverting it onto a *∼*150 μL drop of water. Remove water from the grid by carefully blotting with Whatman Grade 1 qualitative filter paper (cellulose, 11 μm retention size).
(5) PTA staining: proceed with staining by inverting the grid onto a 5 μL drop of 3% PTA, pH 7.5. Stain for 2 min. Remove excess stain with the Whatman paper by blotting and let the grid air-dry.
(6) TEM analysis: store the grids in a clean dry grid-box and analyze by TEM. For assessment of particle shape and integrity, >100,000-fold magnification is preferable.

### Analytical size-exclusion chromatography

Perform size-exclusion chromatography (SEC) to assess the purity of the AAV particles as reported by Gagnon^29^ using a packed-bead Superdex 200 Increase 10/300 GL column at a flow rate of 0.8 mL/min. The sample injection volumes range from 100 to 500 μL.

## Timing

All time points are listed in Table 2.

**Table 2.** Timelines for experimental procedures

Task	Section^a^	Duration
AAV production	3	
Cell seeding and transfection (small scale)	3.1	1 h
Cell seeding and transfection (medium scale)	3.2	≥1 h (depending on plate numbers)
Harvest of AAV particles		30 min +1 h Benzonase treatment
AAV purification		
Sample preparation	3.3	10 min
Column assembly and preparation	3.4	5 min
Purification of AAV particles with a chromatography system	3.5	30 min
Purification of AAV particles manually with a syringe	3.6	15 min
Dialysis (optional) preferably with 300 kDa cutoff	3.7	≥4 h or overnight
Analytics		
Reporter assay for transgene expression	3.8	≈5,000 cells per well for cell seeding; ≈16–24 h for cell attachment (note: it is also possible to directly add vector particles during cell seeding to save time)
Total viral titer determination by PCR	3.9	≥3.5 h
dsDNA assay	3.10	30 min
Total protein assay	3.10	30 min
TEM	3.11	40 min
Analytical SEC	3.12	60 min

dsDNA, double-stranded DNA; SEC, size-exclusion chromatography; TEM, transmission electron microscopy.

^a^ Refer to the following sections in the text: 3, Experimental Procedure; 3.1, AAV Vector Stock Production—Small Scale; 3.2, AAV Vector Stock Production—Medium Scale; 3.3, Sample Preparation for SXC; 3.4, Assembly of SXC Filter Unit; 3.5, SXC Using a Liquid Chromatography System; 3.6, SXC Performed Manually with a Syringe; 3.7, Dialysis of Purified AAV Particles (Optional); 3.8, Reporter Activity Assay to Monitor *In Vitro* Transgene Expression in Cells Transduced with SXC-Purified AAV; 3.9, Determination of AAV Genomes via Droplet Digital™ PCR; 3.10, Evaluation of Impurity Depletion of SXC-Purified AAV Particles; 3.11, Evaluation of Physical Integrity of SXC-Purified AAV by Negative Staining and Transmission Electron Microscopy; 3.12, Analytical Size-Exclusion Chromatography.

## Troubleshooting

Troubleshooting is shown in Supplementary Table S2.

## Results

### Principle of SXC

The mutual PEG exclusion by the cellulose surface and the virus particles favors their association without a direct chemical interaction. Several studies indicate that molecular crowding with PEG in SXC follows many of the rules as precipitation^30–32^: higher PEG concentration and larger PEG size increase the effect, and larger solutes are affected more than smaller ones. This allows the virus particles, which become preferentially hydrated in the presence of PEG, to bind at the surface of the cellulose membrane.^33–35^ Concurrently, impurities with lower hydrodynamic diameter than the virus particles, such as DNA and protein, are washed away, effectively purifying the virus particles. Lee *et al.*^33^ provided an extensive discussion on the variables involved in the performance of SXC. For the theoretical and experimental background on SXC fundamentals and applications, we refer the reader to selected publications.^23,30,31,36,37^

### Product recovery

**Figure 2.**
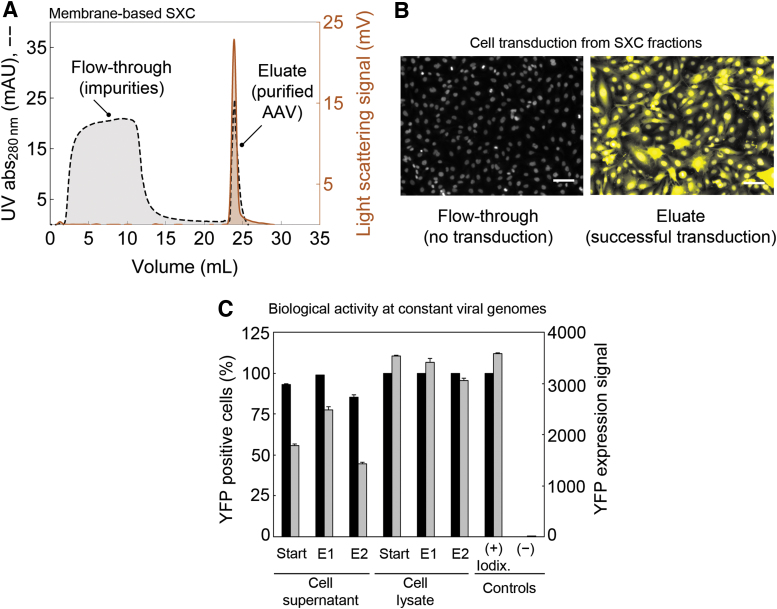
SXC of AAV particles using a single-use filter unit packed with 1.0 μm cellulose membranes. **(A)** Representative SXC chromatogram from a purification of AAV2 particles with a liquid chromatography system. The crude sample was produced as described in the “AAV Vector Stock Production—Small Scale” section and conditioned for SXC as stated in the “AAV Vector Stock Production—Medium Scale” section to 10% PEG-6000. The virus particles were recovered by a step elution to 1 × PBS. The light-scattering signal traces the virus particles. **(B)** Images of the *in vitro* expression of the YFP reporter transgene in SF539 cells treated with either flow-through fractions or elution fractions from SXC-purified AAV2 particles. No YFP expression was detected in the flow-through fractions, indicating no noticeable loss of active AAV particles while loading. Scale bars in panel **(B)** represent 100 μm. **(C)** Biological activity as the *in vitro* expression YFP reporter transgene in U87 cells transduced with AAV-9A2 at roughly 30,000 vg per cell of individual SXC elution fractions (E1 and E2) against an iodixanol-purified sample of the same serotype. Data represent means of analytical replicates (*n* = 3) ± SE. AAV, adeno-associated virus; PEG, polyethylene glycol; SE, standard error; vg, viral genome; YFP, yellow fluorescent protein.

A typical SXC chromatogram is shown in Fig. 2A. As observed in the flow-through, there is no trace of virus particles in the light-scattering signal, whereas the UV signal indicates the depletion of protein, DNA, and other impurities. When the virus particles are eluted from the column by flushing it with a buffer without PEG, the UV and light-scattering signals indicate the presence of AAV particles.

The absence of AAV particles in the flow-through was confirmed by PCR, indicating that 10% PEG-6000 is enough to prevent losses during sample loading. The product recovery by PCR in the elution fractions was 126.1% ± 10.9% (mean ± standard error for serotypes AAV2, AAV6, AAV-1P5, and AAV-9A2; details on the capsid variants have been reported^38^). The estimated yield above 100% is attributed to the error of the PCR assay; similar recovery values have been reported in literature before (*e.g.*, 121%,^39^ or 138%^18^). The high recovery of different AAV particles achieved with the membrane-based SXC protocol described here is consistent with previous SXC results showing nearly full virus particle recovery of different influenza virus (loading at 8% PEG-6000) and yellow fever virus strains (loading at 10% PEG-6000).^25^

### Biological activity

*In vitro* expression of the transgene reporter was used to assess the biological activity of the purified AAV. There was no detectable reporter expression from the flow-through fractions, whereas eluted fractions showed robust expression of the transgene reporter (Fig. 2B). This general trend was observed for several AAV serotypes and peptide display mutants^38^ that were tested, that is, AAV1, AAV2, AAV6, AAV8, AAV-DJP2, AAV-1P5, and AAV-9A2 (transgene reporter signals and transduction ratios from selected AAV types at constant sample volumes used for initial screening of eluates are shown in Supplementary Fig S1).

Figure 2C shows transgene reporter signals and transduction ratios at constant viral genome (vg) levels of SXC eluate fractions against an iodixanol-purified sample. As depicted, virtually, all cells were transduced with both iodixanol- and SXC-purified samples. Expression of the YFP reporter for the cell lysate SXC eluates is comparable to the iodixanol-purified sample, whereas it is lower for the cell supernatant and its SXC eluates. The variation of YFP intensities among the SXC fractions could be due to residual host cell impurities that influence cell transduction efficiency, as reported by Strobel *et al.*^40^ and Tenenbaum *et al.*^41^

**Figure 3.**
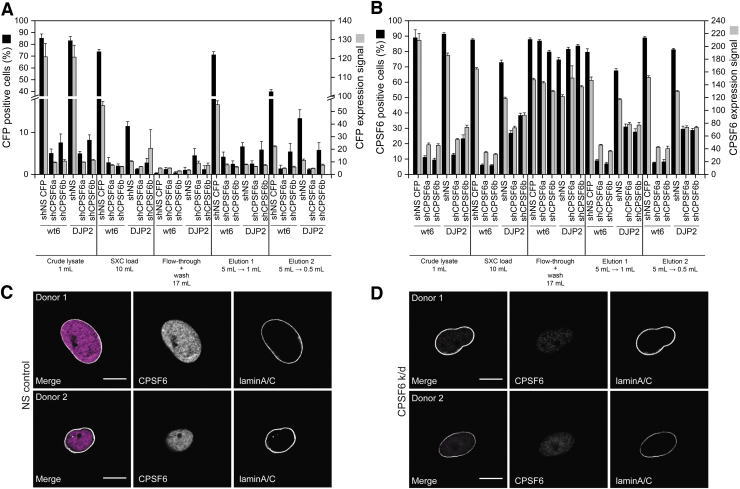
CPSF6 knockdown by transduction with AAV vectors at several stages of their purification with SXC. U87 cells were transduced with different AAV vectors (wt AAV6 and DJP2) encoding a NS shRNA with a CFP reporter or a combination of three anti-CPSF6 shRNAs (shCPSF6a&b). **(A)** CFP-positive cells and CFP expression levels. **(B)** CPSF6-positive cells and CPSF6 expression levels. **(C, D)** Transduction of monocyte-derived macrophages from two different donors with SXC-purified AAV particles (Elution 1 from panels **A** and **B**) carrying an **(C)** NS shRNA or **(D)** three shRNAs targeting CPSF6. Data represent means of analytical replicates (*n* = 18) ± SE. Lamin A/C is a protein marker for the nuclear membrane. Scale bars in cell pictures represent 5 μm. CFP, cyan fluorescent protein; CPSF6, cleavage and polyadenylation specificity factor subunit 6; NS, nonsilencing; wt, wild type.

Besides gene expression, a CPSF6 gene (cleavage and polyadenylation specificity factor subunit 6) knockdown was also evaluated as reported by Bejarano *et al.*^42^ and found to be highly effective (Fig. 3). This further confirms the functionality of the SXC-purified AAV particles for both gene expression and gene knockdown.

### Purity

**Figure 4.**
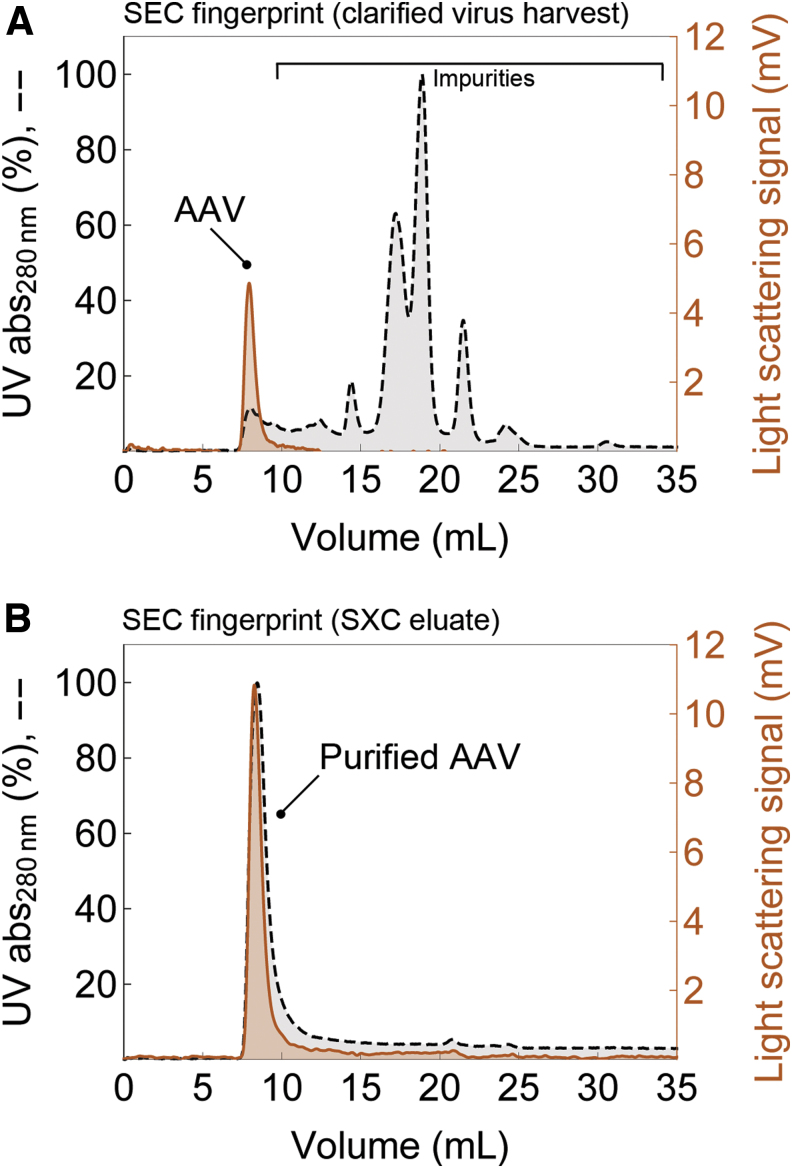
Analytical SEC fingerprints from **(A)** clarified virus harvests before SXC and **(B)** SXC eluates. The profiles show impurities from largest to smallest (*left* to *right*). The AAV particles elute in the column's void volume at around 8 mL without diffusing into the particles' pores due to their large hydrodynamic size compared with smaller impurities. SEC, size-exclusion chromatography.

A SEC analysis of a clarified virus harvest before SXC purification (Fig. 4A) shows the fingerprint of impurities detected by UV absorbance. The light-scattering signal indicates the presence of AAV particles at a retention time of around 8 mL (column's void volume). In contrast, the SEC fingerprint of the SXC-purified AAV particles (Fig. 4B) shows an overlap of the UV and light-scattering signals in the void with no major remaining contaminants of >600 kDa detected by UV absorbance.

**Figure 5.**
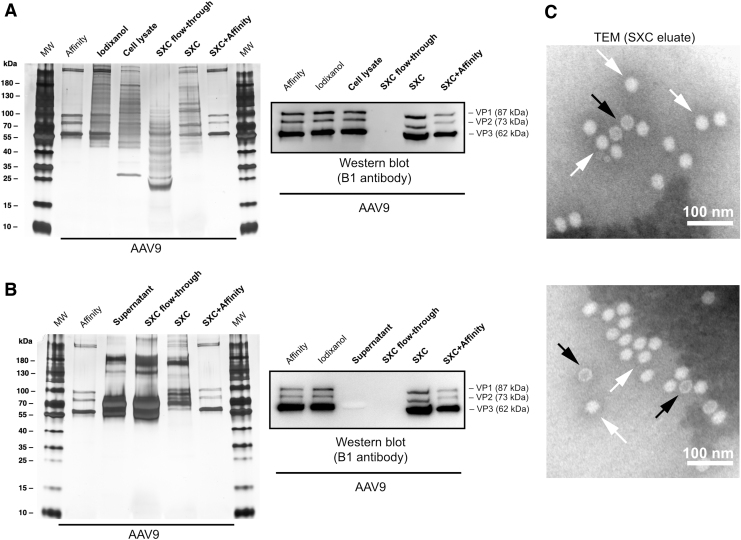
Silver-stained SDS-PAGE and WB of SXC purifications from **(A)** cell lysates and **(B)** cell supernatants of AAV9. Affinity- and iodixanol-purified samples were included as reference. MW is the molecular weight marker. The viral proteins VP1, VP2, and VP3 are indicated. **(C)** TEM of SXC eluates. The AAV particles are homogeneous in shape and size with an approximate diameter of 25 nm. Genome-containing particles (*white arrows*) appear *white* in the negative staining, as opposed to empty capsids (*dark arrows*), which appear as a *white rim with a dark core*. Scale bars represent 100 nm. SDS-PAGE, sodium dodecyl sulfate–polyacrylamide gel electrophoresis; TEM, transmission electron microscopy; WB, Western blot.

Figure 5A and B shows a SDS-PAGE analysis of SXC purifications of recombinant AAV9 in direct comparison to conventional affinity or iodixanol purifications of the identical serotype. As expected, the presence of VP1, VP2, and VP3 was confirmed in all major samples by WB as was their absence in the SXC flow-through fractions, as previously observed by PCR. Notably, the VP1/2/3 bands in the WB of SXC eluates appear to migrate differently as evidenced by their lower apparent molecular weight compared with the VP bands detected in other samples. This is likely an artifact caused by the residual presence of PEG, as previously reported by Arakawa and Gagnon.^43^ Together with this seemingly different band migration behavior, the sample purity did not allow for a unanimous identification of VP1/2/3 in the SXC eluates by silver stain analysis. The SEC fingerprint in Fig. 4B suggests that the protein impurities in the SXC eluates that are detected by silver staining and that hinder the detection of VP1/2/3 bands in this analysis are most probably incorporated into extracellular vesicles. Indeed, the latter have been reported to be co-purified during SXC of virus particles due to their similar size.^22^ A subsequent purification of the SXC eluates with affinity chromatography, which specifically enriches free AAV particles and removes exosomes (Supplementary Fig S2), further confirmed this in the silver stain (Fig. 5A, B). This result highlights both the clearly superior ability of affinity chromatography to remove contaminants as well as its compatibility and synergism with SXC purification.

Importantly, clearance of dsDNA—a major contaminant inevitably occurring in, and confounding, AAV vector production—during SXC is typically >90%.^22,25,33^ For the purification of cell lysates, dsDNA clearance measured by PicoGreen reagent ranged from 94% to 98%. In the case of total protein, the clearance ranged from 80% to 85% measured by the Bradford assay (Supplementary Table S1). These clearance values match previously reported data for SXC of influenza and yellow fever virus particles.^25^

Only 6–18% of AAV particles produced by triple transfection in HEK cells contain the transgene product, and full capsids should be enriched in the final product.^13,14^ Figure 5C shows TEM pictures of crude lysates and SXC-purified AAV particles. The purified virus particles are homogeneous in shape and size with the expected approximate diameter of 25 nm. From the AAV particles observed in Fig. 2E, it seems the percentage of empty AAV capsids in the purified samples is 16–18%. However, these preliminary results should be complemented by additional techniques that provide a more accurate and quantitative characterization of full and empty capsids compared with TEM analysis alone, such as analytical ultracentrifugation, cryo-EM, or multiangle light scattering coupled to refractive index detection.^8,15,44^ The reader is, moreover, referred to the chemistry, manufacturing, and control guidance for human gene therapy drugs published by the U.S. Food and Drug Administration.^45^ Additionally, Penaud-Budloo *et al.* provide a summary on the current testing and specifications for AAV-based products purity and safety.^14^

### SXC device capacity

Using cell lysates, the capacity of the 100 cm^2^ purification device used here was initially ≤2.87 × 10^11^ vg (0.72 × 10^12^ vg/mL) based on the highest titer recovered in the elution pools from cell lysate experiments with no product detected in the flow-through (see Supplementary Table S1 for selected examples). This initially estimated capacity was comparable to values reported by the manufacturers of commercially available products—typically reported at 5–10% of product breakthrough during loading—such as the AVB Sepharose affinity resin (>10^12^ vg/mL).^46^ The manufacturer of the POROS™ AAVX affinity resin reports a binding capacity of >10^14^ vg/mL.^47^ These perfusion particles are rigid and have longer pores that transect the entire particle compared with the shallow dead-end pores of the more traditional AVB Sepharose media.

We hypothesized that the observed capacity of the 100 cm^2^ SXC device used here might be higher based on the observation that no virus particles were found in the flow-through and the fact that membranes and monoliths typically offer 10 to 100 times higher virus particle-binding capacities than porous beads.^29^

We therefore performed additional SXC experiments with a higher AAV challenge to the membrane. It was possible to recover a total of 1.24 × 10^13^ vg in 60 mL of eluate from 830 mL of cell supernatant in around 3.4 h performing four consecutive SXC runs with new SXC 100 cm^2^ devices for every run. Therefore, the calculated capacity of the 100 cm^2^ device was 3.10 × 10^12^ vg, approximately one order of magnitude higher than initially estimated.

### SXC performance

In-line mixing with the PEG stock was preferable compared with the off-line sample conditioning, especially for highly concentrated cell lysates. Adding the concentrated PEG stock to the medium-scale lysates resulted in particle aggregation and hampered sample purification (data not shown). As discussed in more detail by Timasheff^23^ and Lee *et al.*,^33^ in-line mixing with the PEG is encouraged because this way, the target product preferentially interacts with the stationary phase rather than associating with other particles in solution, effectively reducing the risk of aggregation. In-line mixing the PEG is also advantageous because only the right amount of sample is conditioned for the chromatography run.

Elution of the virus particles can be accomplished theoretically in any buffer that does not contain PEG, which is an advantage of SXC over other techniques for AAV purification. Less “harsh” conditions are used for elution compared to, for example, low pH in affinity chromatography, or high salt in IEX, both of which could perturb virus particle stability. Nevertheless, host cell impurities, extracellular vesicles, total particle load, or the isoelectric point (pI, see further discussion below) of the product might modify elution patterns, and slight changes in conductivity during elution might improve product yields if they happen to be lower than expected (Supplementary Table S2).

Besides the hydrodynamic size of the target product, the pI plays an important role in SXC because the amount of PEG needed to achieve binding has a minimum at the pI of the target product when keeping all other parameters constant. Empty and full AAV capsids have different pI values^16^ that might influence binding behavior during SXC, making the separation of full from empty capsids an intriguing and exciting scope for future work.

Overall, our results show that SXC is a promising capture method for purification of AAV particles from cell lysates and cell supernatants using the same recipe regardless of their capsid and surface characteristics. Several AAV types, including multiple wild types, shuffled as well as peptide-displaying AAV variants, were successfully purified by loading at 10% PEG-6000 onto disposable columns packed with regenerated cellulose membranes of 1.0 μm pore size. The average recovery in elution pools from cell lysates was 126.1% ± 10.9% by PCR. The clearance of dsDNA was ≥94%, and the depletion of total protein was ≥80%. The purified AAV particles successfully induced either gene expression or gene knockdown *in vitro*.

The low cost of the membranes allows this operation to be single-use and reduces process development time and cost by eliminating the need of cleaning and sanitization steps. As with other membrane chromatography methods, scale-up is done linearly by increasing the membrane surface. With further research and development, we believe that SXC could be used at industrial scales with spiral wound devices with a membrane surface of around 20 m^2^. Such devices are commercially available (*e.g.*, Sartobind Q Jumbo, 5 L, 8 mm bed height) for other chromatography techniques such as IEX.

Importantly, the high recoveries observed after the initial capture with SXC enable the use of subsequent additional purification steps (*e.g.*, IEX, ultracentrifugation, ultrafiltration/diafiltration to further deplete host cell protein, DNA, empty capsids, and residual PEG) without risking low product recoveries. Moreover, the AAV particles can be loaded and recovered at physiological pH and salt concentration. It is encouraged to load the unpurified samples by in-line mixing with the PEG, as this strategy eliminates the need for sample conditioning beyond clarification.

Finally, the high flow rates that can be used with SXC also offer the possibility of large-scale purification of AAV particles from large volumes of cell supernatants, which is currently a very challenging task with widely used purification methods such as density gradient ultracentrifugation. As we have shown here, using SXC it was possible to recover up to 1.24 × 10^13^ vg in 3.4 h from 830 mL of cell supernatant. In contrast, purifying the same volume using a 1 mL affinity column would take at least 46 h or 15 h of density gradient ultracentrifugation by six rounds of 2.5 h each.

Despite the need for further development of this new technology, we deem membrane-based SXC a versatile and low-cost promising method for the capture of AAV gene transfer vectors.

## Supplementary Material

Supplemental data

Supplemental data

Supplemental data

Supplemental data
