# Supplementary material for: Single-Use Capture Purification of Adeno-Associated Viral Gene Transfer Vectors by Membrane-Based Steric Exclusion Chromatography
Source: Hum Gene Ther. 2021 Sep 23;32(17-18):959–74. doi: 10.1089/hum.2019.284 (PMC10116406; doi:10.1089/hum.2019.284)
Supplement: Supplemental data [file Supp_TableS2.pdf]

Supplementary Table ST2. Troubleshooting.

Issue	Possible reason(s)	Solution
AAV production		
Cell seeding and transfection	Poor transfection ratio or cell death due to wrong cell numbers	Optimize cell density
AAV purification		
Filter clogging while filtering sample	Too many submicron-sized impurities	Centrifuge the virus harvest at >8000 rcf and use a cascade of filters for clarification, *e.g.*, 0.8 µm, 0.45 µm, and 0.2 µm
Turbidity when mixing sample with PEG	Virus aggregation	Mix the PEG with the sample in-line with a chromatography system. See Section 3.5.1
	Virus aggregation or impurity aggregation	Dilute the sample or screen different buffers for increased colloidal stability (*e.g.*, spiking with sugars such as sucrose or sorbitol)
Pressure and UV spikes during chromatography run	Air in the UV cell of the chromatography system	Degas buffers and remove the air from the system. See equipment manual
No UV signal during elution or product found in the flow-through	Low concentration of virus particles in the elution	Ensure the PEG concentration is 10% for loading. Lower PEG concentrations might result in product loss during sample loading
Low product yields or multiple peaks during elution	Overloaded membrane (fouling), presence of extracellular vesicles or incomplete elution of AAV particles due to host cell impurities or isoelectric point profiles	Fractionate elution to screen for the presence of the AAV particles or perform a gradient elution instead of a step to improve resolution.<br>Test different elution conditions at higher/lower conductivities to disrupt the interactions of AAV particles or impurities due to non-specific interactions or isoelectric points (see discussion in text)
Analytics		
Variable cell transduction from different elution fractions	Variable potency due to AAV concentration or presence of host cell impurities	Perform cell transduction at constant viral genome levels. The presence of host cell impurities might influence cell transduction *in vitro* (see discussion in text)
Stain precipitates on the grid (TEM)	Precipitation of uranyl phosphate due to the use of PBS	Use phosphotungstic acid (PTA) instead of uranyl acetate
		Dialyze the sample overnight to a Tris-based buffer in order to eliminate the PBS
Grid overstaining or film breakage (TEM)	Overloading of the grid with product	Dilute the sample
