# Supplementary material for: Single-Use Capture Purification of Adeno-Associated Viral Gene Transfer Vectors by Membrane-Based Steric Exclusion Chromatography
Source: Hum Gene Ther. 2021 Sep 23;32(17-18):959–74. doi: 10.1089/hum.2019.284 (PMC10116406; doi:10.1089/hum.2019.284)
Supplement: Supplemental data [file Supp_FigS1.pdf]

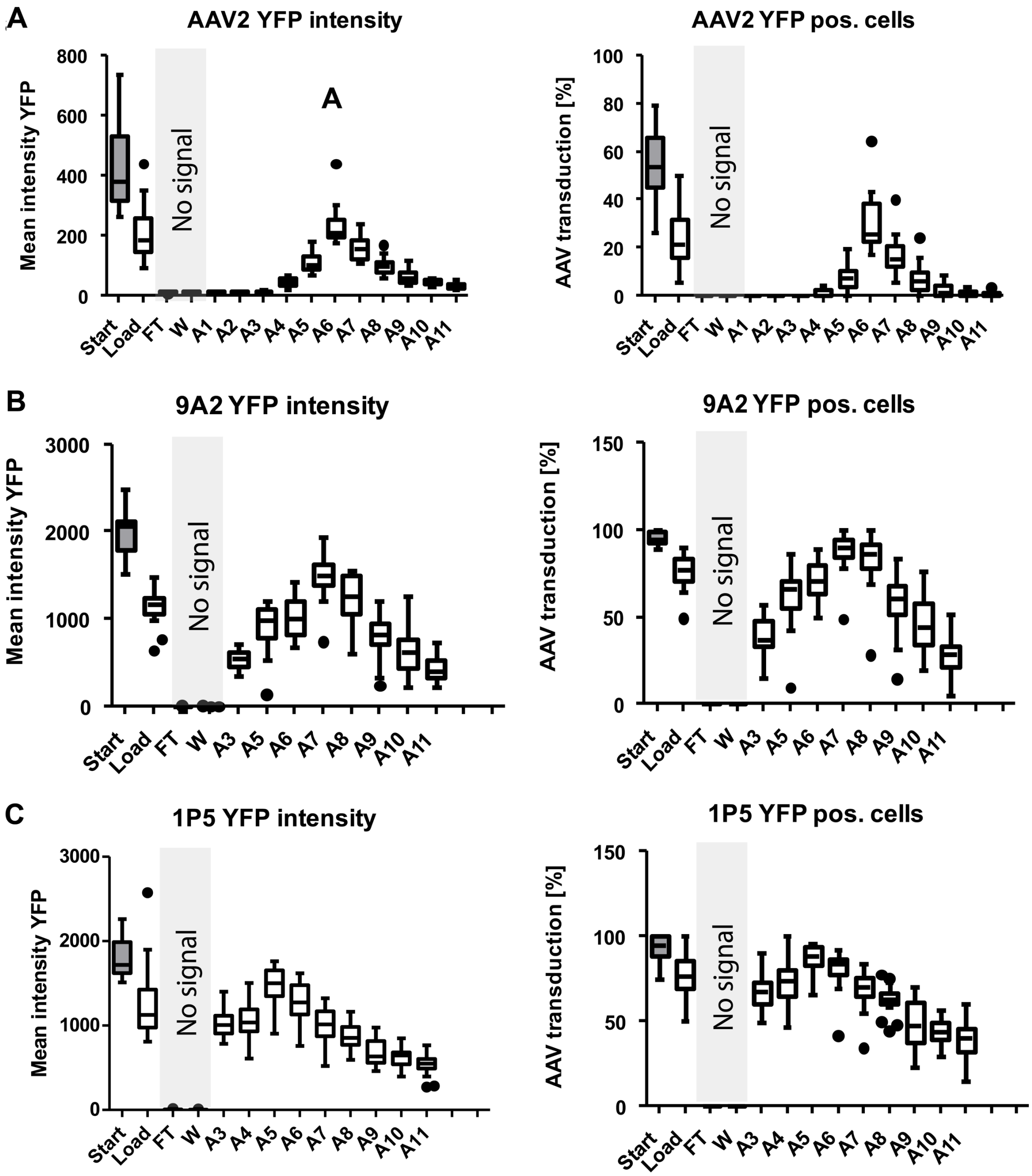

Supplementary Figure SF1. Transgene reporter expression levels and reporter-positive cells. U87 cells were transduced with vectors based on selected AAV capsid variants and purified by SXC. Start=unpurified crude lysates; Load=PEG-conditioned sample before SXC; FT=flow-through; W=wash; AX=elution fractions. Microscopy-based readout was used to quantify the transduction rate based on YFP-positive cells (left panel) and the mean intensity of YFP fluorescence (right panel). Results are depicted for three capsid variants, i.e., the AAV prototype AAV2 (top), as well as synthetic AAV capsids 9A2 (middle) and 1P5 (bottom). The dots are outliers (less than 3/2 times of lower quartile or more than 3/2 of upper quartile, respectively).
