# Supplementary material for: Single-Use Capture Purification of Adeno-Associated Viral Gene Transfer Vectors by Membrane-Based Steric Exclusion Chromatography
Source: Hum Gene Ther. 2021 Sep 23;32(17-18):959–74. doi: 10.1089/hum.2019.284 (PMC10116406; doi:10.1089/hum.2019.284)
Supplement: Supplemental data [file Supp_FigS2.pdf]

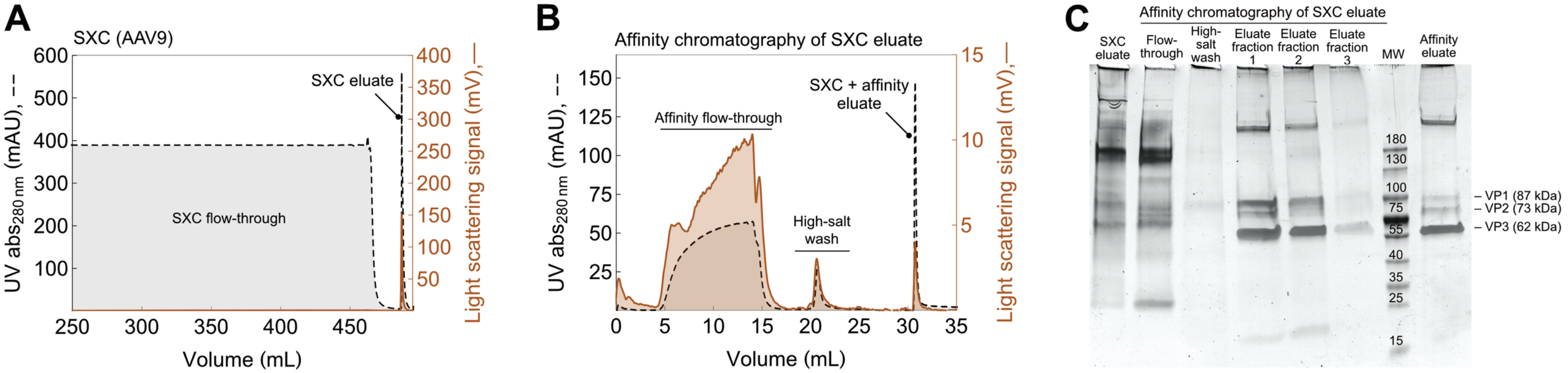

Supplementary Figure SF2. (A) SXC of AAV9, (B) subsequent affinity chromatography of SXC eluate, and (C) corresponding SDS-PAGE silver stain of the purification fractions. The light scattering signal traces nanoplexes such as virus particles and extracellular vesicles. Silver stain of the SXC eluate shows multiple protein bands in the range of 25–200 kDa that are most probably incorporated into extracellular vesicles as evidenced by SEC analysis which shows no major impurities in the SXC eluate of less than 600 kDa (see Figure 4). These remaining impurities are successfully eliminated by affinity chromatography.
