# Supplementary material for: Single-Use Capture Purification of Adeno-Associated Viral Gene Transfer Vectors by Membrane-Based Steric Exclusion Chromatography
Source: Hum Gene Ther. 2021 Sep 23;32(17-18):959–74. doi: 10.1089/hum.2019.284 (PMC10116406; doi:10.1089/hum.2019.284)
Supplement: Supplemental data [file Supp_TableS1.pdf]

Supplementary Table ST1. Product recoveries, total protein, and host cell DNA balances for medium-scale lysates of selected AAV serotypes and recombinant variants purified with membrane-based SXC.

			Virus product			Impurities					
			PCR			Total Protein			dsDNA		
**Product**	**SXC step**	**Vol (mL)**	**vg/mL**	**vg**	**%**	**µg/mL**	**µg**	**%**	**ng/mL**	**ng**	**%**
AAV2	Start (before PEG conditioning)	9.5	1.01E+10	9.57E+10	100.0	827.6 ± 1.2	7,861.8 ± 11.7	100.0	9,031.8 ± 100.8	85,802.1 ± 957.6	100.0
	Flow-through+wash	40.0	0.00E+00	0.00E+00	-	139.9 ± 0.8	5,597.6 ± 33.6	71.2 ± 0.4	n.d.	n.d.	n.d.
	Eluate	20.0	6.98E+09	1.40E+11	145.9	60.1 ± 0.5	1,201.5 ± 9.5	15.3 ± 0.1	264.6 ± 12.2	5,291.0 ± 244.5	6.2 ± 0.3
AAV6	Start (before PEG conditioning)	9.5	9.70E+09	9.21E+10	100.0	836.7 ± 6.4	7,948.7 ± 60.8	100.0	14,137.7 ± 100.5	134,308.2 ± 954.8	100.0
	Flow-through+wash	45.0	0.00E+00	0.00E+00	-	141.2 ± 1.2	6,351.8 ± 54.5	79.9 ± 0.9	n.d.	n.d.	n.d.
	Eluate	18.5	4.91E+09	9.33E+10	101.3	83.1 ± 0.7	1,605.4 ± 13.0	20.2 ± 0.2	161.0 ± 12.1	3,157.1 ± 235.5	2.4 ± 0.2
AAV-1P5	Start (before PEG conditioning)	9.5	1.96E+10	1.86E+11	100.0	824.1 ± 4.3	7,829.0 ± 40.9	100.0	10,106.9 ± 51.1	96,015.6 ± 485.5	100.0
	Flow-through+wash	45.0	0.00E+00	0.00E+00	-	139.0 ± 0.7	6,252.8 ± 29.3	79.9 ± 0.6	n.d.	n.d.	n.d.
	Eluate	18.6	1.12E+10	2.12E+11	114.0	85.3 ± 2.3	1,639.9 ± 44.7	20.9 ± 0.6	92.7 ± 9.5	2,034.1 ± 239.7	2.1 ± 0.2
AAV-9A2	Start (before PEG conditioning)	9.5	2.11E+10	2.01E+11	100.0	879.8 ± 3.3	8,358.1 ± 31.4	100.0	11,324.3 ± 51.8	107,580.9 ± 492.1	100.0
	Flow-through+wash	45.0	0.00E+00	0.00E+00	-	145.4 ± 0.7	6,541.7 ± 33.3	78.3 ± 0.5	n.d.	n.d.	n.d.
	Eluate	18.6	1.51E+10	2.87E+11	143.0	88.7 ± 1.0	1,706.2 ± 20.3	20.4 ± 0.3	92.7 ± 12.3	1,797.7 ± 240.8	1.7 ± 0.2
	Mean of eluates				126.1 ± 10.9			18.6 ± 0.2			3.4 ± 0.2

n.d.; not determined
